# Krill-Oil-Dependent Increases in HS-Omega-3 Index, Plasma Choline and Antioxidant Capacity in Well-Conditioned Power Training Athletes

**DOI:** 10.3390/nu13124237

**Published:** 2021-11-25

**Authors:** Franchek Drobnic, Andreas B. Storsve, Lena Burri, Yunpeng Ding, Montserrat Banquells, Joan Riera, Per Björk, Ventura Ferrer-Roca, Joan Carles Domingo

**Affiliations:** 1 Medical Services Shanghai Shenhua FC, Shanghai 201315, China; docdrobnic@gmail.com; 2 Aker BioMarine Antarctic ASA, 1366 Lysaker, Norway; storsve@gmail.com (A.B.S.); yunpeng.ding@akerbiomarine.com (Y.D.); 3 Departamento de Fisiología del Deporte, Centro de Alto Rendimiento (CAR), 08174 Sant Cugat del Vallés, Spain; mbanquells@car.edu (M.B.); joanrieracanals@gmail.com (J.R.); 4 Direction and innovation Dept, Cienporciennatural, 28035 Madrid, Spain; per@cienporciennatural.com; 5 Departamento de Biomecánica del Deporte, Centro de Alto Rendimiento (CAR), 08174 Sant Cugat del Vallés, Spain; vferrer@car.edu; 6 Departamento de Bioquímica y Biología Molecular, Facultad de Biología, Universidad de Barcelona, 08028 Barcelona, Spain; jcdomingo@ub.edu

**Keywords:** choline, CrossFit^TM^, power training, DHA, EPA, high-intensity interval training, krill oil, HS-Omega-3 Index, oxidative stress, phosphatidylcholine, sports nutrition

## Abstract

There is evidence that both omega-3 polyunsaturated fatty acids (n-3 PUFAs) and choline can influence sports performance, but information establishing their combined effects when given in the form of krill oil during power training protocols is missing. The purpose of this study was therefore to characterize n-3 PUFA and choline profiles after a one-hour period of high-intensity physical workout after 12 weeks of supplementation. Thirty-five healthy power training athletes received either 2.5 g/day of Neptune krill oil^TM^ (550 mg EPA/DHA and 150 mg choline) or olive oil (placebo) in a randomized double-blind design. After 12 weeks, only the krill oil group showed a significant HS-Omega-3 Index increase from 4.82 to 6.77% and a reduction in the ARA/EPA ratio (from 50.72 to 13.61%) (*p* < 0.001). The krill oil group showed significantly higher recovery of choline concentrations relative to the placebo group from the end of the first to the beginning of the second exercise test (*p* = 0.04) and an 8% decrease in total antioxidant capacity post-exercise versus 21% in the placebo group (*p* = 0.35). In conclusion, krill oil can be used as a nutritional strategy for increasing the HS-Omega-3 Index, recover choline concentrations and address oxidative stress after intense power trainings.

## 1. Introduction

The high-intensity modality of exercise performed during short periods of time has gained popularity in the last decades due to factors related to health, fashion and a sense of well-being. These activities are known as power training (PT) sports, or high-intensity interval training activities (HIIT), but sometimes popularly referred to as CrossFit, which is a registered trademark (CrossFit^TM^). Whereas power lifting focuses on bench press, squat and deadlift, the PT schedule is characterized by functional movements performed at high intensity, combining intervals of strength and endurance that can vary between weightlifting, running, cycling, squatting, pulling, pushing, etc. with little or no rest in between [1,2]. Because this functional movement training method is metabolically very demanding, its beneficial effect on health and on improving athletic performance has been contradictorily assessed [3,4,5,6]. Since risks for prolonged responses of oxidative stress and inflammation as well as injury and overreaching have been highlighted, dietary interventions may be required to improve athletic performance and speed up recovery. There are some studies available that evaluated the benefits of nutritional interventions in PT athletes [7,8,9,10,11,12,13]. However, no interventional data exist that establish choline and omega-3 polyunsaturated fatty acid (n-3 PUFA) intake as a supplementation strategy to optimize PT training sessions and recovery, and these important nutrients seem so far to go unnoticed in this sporting discipline [14]. Given that 90% of the US population is known to consume too little choline [15], and that European populations also do not reach the recommended daily intake levels of 400 mg [16], this indicates that athletes might also be at risk of choline deficiency. The body can produce choline in limited amounts by using one-carbon groups from the folate metabolism, but most of it has to be supplied by the diet [17]. It is noteworthy that a person’s need for endogenous choline is increased if genetic modifications of genes involved in the folate metabolism are present, which will further increase the risk for choline deficiency [18].

During exercise, plasma choline is needed as a precursor for acetylcholine, which is a neurotransmitter responsible for muscles contractions [19,20,21]. If not enough plasma choline is available, then the body can resort to breaking down phosphatidylcholine, the building blocks of cell membranes, which can compromise membrane integrity and stress resistance [22,23]. Vulnerable membranes and reduced acetylcholine production during intense physical exercise might promote muscle damage and decrease muscle stimulation resulting in muscle exhaustion [17]. In addition to being essential for neurotransmitter synthesis, choline is involved in cell-membrane signaling, transport of fat and methyl group metabolism, which are all important functions for optimal sports performance [24]. However, plasma choline concentrations are known to be challenged during strenuous physical exercise. In particular, in endurance athletes performing more than two hours of intense, physical activity at more than 70% VO_2max_, a significant decrease of plasma choline concentrations has been described [17]. In both marathon runners [20,25] and cyclists [26], the absence of choline supplementation can lead to a 40% reduction in plasma choline, which might challenge cellular functions and limit performance. Evidence that confirms whether the availability of choline is also compromised during power challenges such as PT, and whether choline supplementation might benefit this sporting discipline, is scarce [17]. One study with college-aged males described supplementation with daily 600 mg alpha-glycerylphosphorylcholine and the effect on increasing lower- and upper-body isometric strength production [27]. After six days of supplementation, the athletes had significant gains in lower-body strength when performing isometric mid-thigh pulls on a force plate, an exercise linked to weightlifting performance.

A sustainable source of choline is provided by krill oil extracted from Antarctic krill (*Euphausia superba*). In krill oil, choline is found in the form of phosphatidylcholine [28], which was shown to significantly increase plasma choline levels and some of its metabolites in a single 8 g dose plasma kinetic study in healthy volunteers [29]. A longer study over four weeks confirmed that 4.5 g daily krill oil administration significantly increases plasma choline and betaine concentrations in healthy young adults [30]. In an athletic setting, notably after the Ironman-distance Norseman Xtreme triathlon, serum choline concentrations significantly decreased by 34% from pre- to post-race [31]. On the other hand, 4 g of krill oil given for 5 weeks before the race significantly increased serum choline levels both before and after the race when compared to the placebo group.

Krill oil, however, not only provides choline, but also n-3 PUFAs that are bound to the phospholipid molecules. In particular, the n-3 PUFAs eicosapentaenoic acid (EPA; C20:5 n-3) and docosahexaenoic acid (DHA; C22:6 n-3) have been intensively studied for their immunomodulatory, anti-inflammatory and pro-resolving benefits [32,33,34,35]. Since high-intensity training has been discussed to negatively impact immune function and inflammation [36], n-3 PUFA supplementation may provide a mean to address dietary deficiencies and improve athletic recovery and resistance to infection [37]. Indeed, benefits for post-exercise immune function after 2 g/d of krill oil for six weeks [38] have been demonstrated. Moreover, EPA and DHA are known to stimulate muscle protein anabolism [39] and 3 g of krill oil given for eight weeks to resistance-trained subjects was shown to activate mTOR signaling, which is known to trigger an increase in muscle mass [40]. 

A mean to test EPA and DHA levels is given in the Omega-3 Index and standardization of the analytical procedure is ensured for the HS-Omega-3 Index [41]. The HS-Omega-3 Index is defined by the EPA and DHA concentrations as a percentage of all red blood cell (RBC) fatty acids (FAs), which has been suggested to best represent long-term n-3 PUFA intake and general n-3 tissue status, in particular atrial levels [42]. EPA and DHA become biologically important once they are integrated into membranes, hence the analysis of RBC membranes provides a convenient mean to assess tissue distribution. Most importantly, the HS-Omega-3 Index has been recognized as a risk factor for sudden cardiac death and an increased risk for cardiovascular disease has been described when the HS-Omega-3 Index is ≤4%, an intermediate risk from >4% to <8% and a low risk if ≥8% [34]. It is therefore recommended to reach a target range of 8–11% to lower the risk for disease [43]. This can be achieved by consuming fatty fish or marine n-3 dietary supplements that provide long-chain n-3 PUFAs [44,45], since conversion rates from the short-chain n-3 alpha-linolenic acid (ALA) to EPA and DHA in humans are rather poor (8–20% for ALA to EPA; 0.5–9% for ALA to DHA) [46]. 

Besides their anti-inflammatory effects, n-3 PUFAs are also known to have anti-oxidant properties that might deal with the reactive oxygen species (ROS) that are generated proportionally to the intensity of the physical activity [47,48]. This exercise-induced oxidative stress can contribute to acute muscle fatigue [49], but at the same time is important for cellular signaling to adapt to training and induce an antioxidant defense response [50]. The antioxidant defense system in the body includes both endogenous enzymatic and non-enzymatic antioxidant defenses, as well as endogenous antioxidants supplied by the diet. Exercise duration, intensity, fitness condition and nutritional status of the athlete will define if the level of ROS produced is helpful or harmful [51]. Chronic exposure of high ROS levels can be harmful, exhaust the antioxidant defense systems and result in oxidative damage and impaired cellular function. Nutritional interventions can therefore be of interest to reduce oxidative stress, decrease muscle soreness and improve sports performance. So far it has been shown that 1 g/d of krill oil supplementation for six weeks reduced oxidative stress in professional rowers submitted to exhaustive exercise [52]. In a study with coronary heart disease patients that received 2 g/d krill oil for three months, it was proposed that antioxidant capacities were increased via the Kelch-like ECH-associated protein 1-NF-E2-related factor 2 (KEAP1-NRF2) signaling pathway [53].

In addition to n-3 PUFAs, antioxidant help might also be found in the form of the carotenoid pigment astaxanthin (3,3′-dihydroxy-β,β′-carotene-4,4′-dione) that krill oil contains with both hydroxyl groups esterified to FA [40]. Astaxanthin has thirteen conjugated double bonds and because of their arrangement, astaxanthin has strong antioxidant properties [54]. The astaxanthin dose that 2.5 g of krill oil provides is around 1.7 mg, which is below the recommended dose of 4 mg for athletes that is linked to improved muscle damage, time trial performance and power output [55,56,57]. Nevertheless, it has been suggested that the phospholipids of krill oil may increase intestinal absorption of astaxanthin [40], thereby optimizing its availability to the body for integration into cell membranes and fight against excessive free radical production in athletes [53].

As a result, this 12-week study intended to assist in laying the foundation for optimal performance and recovery of PT athletes by demonstrating the effect of krill oil on HS-Omega-3 Index levels and plasma choline recovery after exercise, as well as on scavenging free radicals after high-power physical workouts.

## 2. Materials and Methods

### 2.1. Participants and Procedures

Out of 95 screened subjects, 36 individuals fitted the inclusion criteria and agreed to take hard gelatin capsules (Licap^®^ encapsulation) at the prescribed dose for 12 weeks prior to a training session. The nutritional composition of the study products is given in Table 1.

One subject was non-compliant (showing a decrease in the HS-Omega-3 Index after 12 weeks of krill oil supplementation) and therefore was excluded, leaving a total of 35 subjects (27 males and 8 females) in the final sample. A total of 19 participants were included in the krill oil and 16 participants were included in the placebo group (Table 2).

### 2.2. Eligibility Criteria and Follow-Up

Eligibility criteria were based on selecting healthy athletes of both sexes, between the ages of 21–40 who had a HS-Omega-3 Index of less than 6% and were in good health, without chronic or acute inflammatory pathology and normal blood parameters. Volunteers were excluded when they had had a muscle injury within the last 6 months or used any sort of medication. Moreover, the athletes had to be practicing the sporting discipline of PT (or any similar activity such as CrossFit™) in a sports gym with a specialized instructor for at least 3 years in a row at least four days per week. Another inclusion criterion was the commitment of the participants to avoid the use of any nutritional supplements and stay on their normal diet during the study and 15 days prior to study start. To this end, various clubs were asked for volunteers to participate. A total of 95 medical examinations were carried out with cardiometabolic-monitored maximal exercise stress tests, blood standard analyses and determination of the HS-Omega-3 index in order to reach the number considered appropriate for this pilot study (*n* = 40).

During the study, subjects were reminded every week by email of their commitment to adhere to the study and every 30 days they had to come to the Centre d’Alt Rendiment (CAR), Olympic Training Center (OTC) of Barcelona, Spain to obtain the supplementation for the following month. After two months from trial inclusion, a blood sample was obtained to assess compliance.

The COVID epidemic was a major hindrance for the recruitment of the last group of study subjects, nevertheless, there were 18 athletes who voluntarily wanted to conclude the study and prolonged the supplementation with krill oil (*n* = 5) or placebo (*n* = 13) for another month until the analytical evaluations were carried out. The CAR health administration allowed a baseline blood draw in these 18 athletes, but because of the epidemic, the training sessions to evaluate the scavenging free radicals after a high-power physical workout could not be carried out.

### 2.3. Exercise Training Sessions Protocol

In the interest of performing a similar training session, typical of a PT facility, the same instructor performed all sessions with the same exercise model with the same progression and structure. Six to eight individuals participated in the sessions that were always performed at the same time in the day, i.e., in the morning, with the same breakfast and dinner the day before the session. The training sessions were scheduled on a Thursday, having done moderate training on Monday and Tuesday and no training on Wednesday. That means that the athletes had at least 24 h of rest from the last training session, which was at low intensity. The work session protocol included 10 min of oriented warm-up, and then two series with various stations of intense work lasting 20 min with 10 min of rest in between. Heart rate was monitored in each athlete using the Polar RS800CXTM HR monitor. At the end of each series, lactate was measured by micromethod in arteriolarized blood (lactate analyzer LACTATE PRO 2, Arkray KDK, Japan) at 3 and 5 min and the internal load session rating of perceived exertion (RPE) was assessed through the Borg CR-10 scale adapted from [58].

### 2.4. Blood Sample Collection and Analysis of Total Antioxidant Capacity 

At baseline, the samples for the standard analysis and HS-Omega-3 Index were collected during the fasting state. A 20 mL blood sample was taken by venipuncture, processed and stored for analysis of the HS-Omega-3 Index and standard health parameters at baseline, the HS-Omega-3 Index two months after study start and at the end of the supplementation period, as well as the total antioxidant capacity (TAC) and malonyl dialdehyde (MDA) concentrations before and after the training session.

The TAC in plasma samples was measured using the OxiSelect™ total antioxidant capacity assay kit (STA-360, Cell Biolabs Inc., San Diego, CA, USA) following the manufacturer’s instructions. High values in the TAC assay reflect a high antioxidant capacity, i.e., greater protection. Uric acid equivalent was used to calculate µM copper-reducing equivalent values. The level of MDA was measured colorimetrically using the OxiSelect™ TBARS assay kit (MDA Quantitation, STA-330, Cell Biolabs Inc., San Diego, CA, USA). MDA quantitation results are expressed as µmol/L (µM) concentration using the MDA standard curve obtained during the processing of the plasma samples. 

Body composition was assessed by kinanthropometry following international standards for anthropometric assessments applying the Yuhasz formula modified by Faulkner [59,60].

### 2.5. HS-Omega-3 Index Analysis

Blood was collected into a vacutainer containing EDTA, and red blood cells (RBCs) and plasma were separated by centrifugation at 3000 rpm for 15 min at room temperature and kept on dry ice until stored at −80 °C. The RBCs were used for the analysis of the membrane FA composition at Omegametrix GmbH (Martinsried, Germany). In short, FA methyl esters from RBCs were identified by gas chromatography (GC2010, Shimadzu, Duisburg, Germany) equipped with a SP2560 100 m column (Supelco, Bellefonte, PA, USA) using hydrogen gas as a carrier by comparison with a known standard (GLC-727; Nuchek Prep, Elysian, MN, USA). The HS-Omega-3 Index was given as EPA + DHA in RBCs expressed as a percentage of the total identified FAs.

### 2.6. Choline Analysis

The plasma was used for analysis of free choline and its metabolites at the laboratory of Bevital AS (Bergen, Norway). Measurements were carried out using an 1100 HPLC system (Agilent Technologies, Santa Clara, CA, USA). The HPLC system was coupled to an API3000 triple-quadrupole tandem mass spectrometer (AB Sciex, Framingham, MA, USA) equipped with an electrospray ion source and fitted with a hot-source-induced desolvation from IONICS (Calamba City, Philippines). Analyst (Ver.1.5.2; AB Sciex, Framingham, MA, USA) was used for data acquisition and analysis. Sample processing was performed by a MikroLab AT Plus robotic workstation (Hamilton, Bonaduz, Switzerland) and samples of 10 μL of deproteinized plasma were injected onto a Fortis phenyl column from Fortis Technologies Ltd. (Cheshire, UK) guarded by a Polar-RP SecurityGuard cartridge (Phenomenex, Torrance, CA, USA). The mass spectrometer was operated in the positive ESI mode, and analytes and internal standards were detected in MRM with unit resolution at quadrupole 1 (Q1) and low resolution at Q3. More details are given elsewhere [61].

### 2.7. Statistics

Data were analyzed using IBM SPSS statistics (v27). An independent samples t-test was used to test baseline group differences as well as between-group differences in FA profiles. Paired sample t-tests were used to test for within-group changes in individual FAs across the supplementation interval. Repeated measures three-way ANOVA was employed to test for overall and interaction effects of time point (pre-supplementation, post-supplementation; within-subject factor), exercise (pre-exercise, post-exercise; within-subject factor) and supplement type (krill oil and placebo, between-subject factor) on RBC FA profiles, including HS-Omega-3 Index, choline concentration, and TAC status. The assumption of sphericity was tested with Mauchly’s test of sphericity, and adjusted *p*-values are given where this assumption is violated. Alpha was set at 0.05.

## 3. Results

No group differences were found for any of the baseline characteristics described in Table 3 (all *p* values > 0.05).

### 3.1. HS-Omega-3 Index

**Figure 1:**
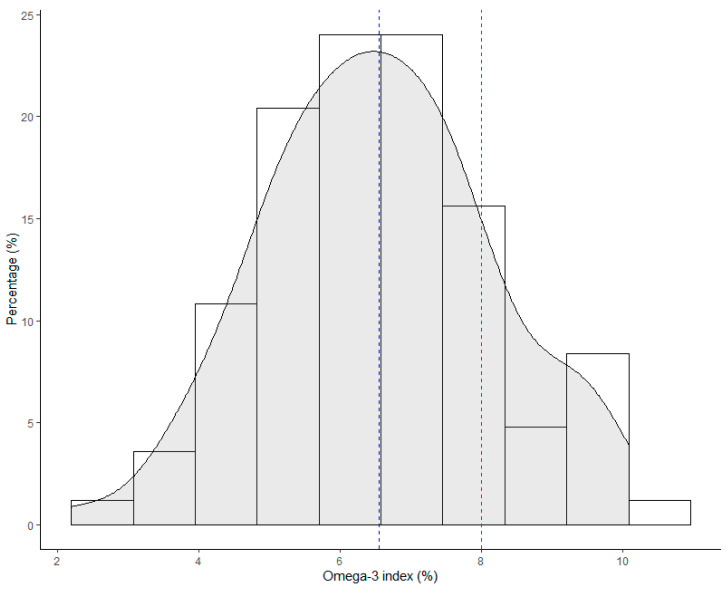
The distribution of the HS-Omega-3 Index values among athletes at screening. The red line indicates the threshold of optimal HS-Omega-3 Index (8%), while the blue line indicates the mean value of HS-Omega-3 Index (6.55%).

During recruitment, 95 athletes were screened for the HS-Omega-3 Index. The mean value (±SD) was 6.55% (±1.60%) (Figure 1). Among these subjects, the lowest HS-Omega-3 Index was 2.2%, while the highest was 10.1%. Notably, only 17.9% of the subjects (*n* = 17) had a HS-Omega-3 Index above or equal to 8%. In addition, there were 37.9% of the subjects (*n* = 36) with a HS-Omega-3 Index lower than 6% and 5.2% of the subjects (*n* = 5) were with a HS-Omega-3 Index value below 4%. The proportion of subjects with a HS-Omega-3 Index between 4 and 8% was 76.8% (*n* = 73). 

**Figure 2:**
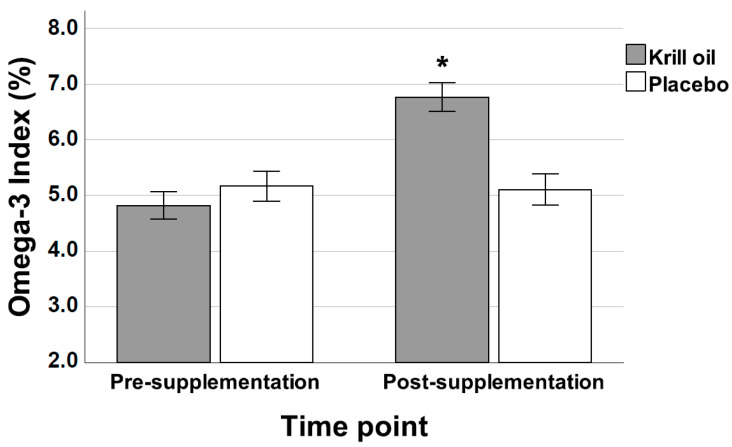
Mean (±SE) HS-Omega-3 Index across the study period of 12 weeks for the krill oil and placebo groups. * indicates a significant group difference (*p* < 0.05).

Figure 2 shows group-dependent changes in the HS-Omega-3 Index, with only the krill oil group showing an increase across the study period. A two-factor mixed-design ANOVA revealed a significant effect of time point (F1,33 = 55.3, *p* < 0.001) and a significant time point × supplement type interaction effect (F1,18 = 27.61, *p* < 0.001). An inspection of Figure 2 suggests that this interaction effect is due to the krill oil but not the placebo group, showing a pronounced increase in HS-Omega-3 Index levels following supplementation. Post-hoc analyses confirmed this interpretation of the data. The krill oil group had a mean increase in the HS-Omega-3 Index from 4.82 to 6.77% across the supplementation period, which was significant (t(18) = 10.5, *p* < 0.001). 

### 3.2. Individual Fatty Acids

Table 4 shows RBC membrane FA composition before and after supplementation. FA profiles remained stable across the supplementation period in the placebo group. However, in the krill oil group there were significant increases in the n-3 PUFAs EPA (from 0.41 to 1.34%; *p* < 0.001), DPA (from 1.60 to 2.46%; *p* < 0.001) and DHA (from 4.41 to 5.31%; *p* < 0.001), as well as a significant decrease in the n-6 PUFA arachidonic acid (ARA) (from 0.41 to 1.34%; *p* = 0.006). Furthermore, significantly higher post-supplementation values were found for EPA (*p* < 0.001), DPA (*p* < 0.001) and DHA (*p* < 0.05) and significantly lower values for ARA (*p* < 0.01) in the krill oil group vs. the placebo group.

### 3.3. Sum of Fatty Acids

An inspection of Table 4 shows that there were no significant changes in trans FAs, saturated FAs or monounsaturated FAs for any of the groups. However, a significant increase in n-3 FAs (from 6.51 to 9.18%; *p* < 0.001) were found for the krill oil group, as well as a significant reduction in n-6 FAs (from 35.76 to 33.42%; *p* < 0.001), n-6/n-3 ratio (from 5.84 to 3.77; *p* < 0.001) and ARA/EPA ratio (from 50.72 to 13.61; *p* < 0.001). No within-group changes were observed for the placebo group on the same parameters. Furthermore, the krill oil group showed significantly higher post-supplementation values relative to the placebo group for n-3 FAs (*p* < 0.001), and significantly lower post-supplementation values for n-6 FAs (*p* < 0.001), n-6/n-3 ratio (*p* < 0.001) and ARA/EPA ratio (*p* < 0.001).

### 3.4. Exercise Training Session Load

The results of the physiological internal load and subjective effort made are given in Table 5. An expected and desired work intensity was observed, similar to what is seen in a normal PT session [1]. No statistical difference in work intensity was observed between the two treatment groups, nor between sessions of the same group, thus indicating that the work pattern was the same for all four PT sessions.

### 3.5. Choline

**Figure 3:**
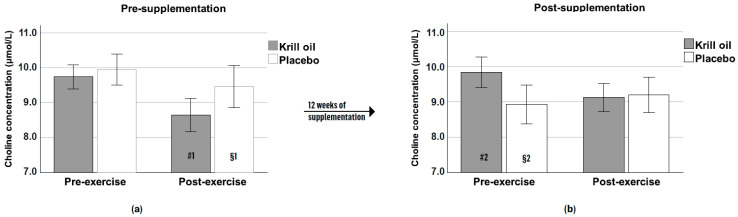
Mean (±SE) plasma choline concentration (µmol/L) pre- (**a**) and post-supplementation (**b**) for the krill oil and placebo groups. # and § depict values used for a follow-up *t*-test for Figure 4.

Complete choline data sets were available from a subsample of 21 participants. An inspection of Figure 3a reveals pre-supplementation decrements in choline levels from pre- to post-exercise in both groups (from 9.7 to 8.6 µmol/L in the krill oil group and 9.9 to 9.4 µmol/L in the placebo group), with a similar pattern also being shown in the krill oil group post-supplementation (Figure 3b). A mixed-design repeated measures ANOVA supports this interpretation of the data, revealing a significant main overall effect of exercise (pre-exercise; post-exercise) (F1,19 = 7.19, *p* = 0.015) across two time points (pre-supplementation; post supplementation), and a significant overall exercise × time point interaction effect (F1,19 = 3.17, *p* = 0.05) in the trial.

**Figure 4:**
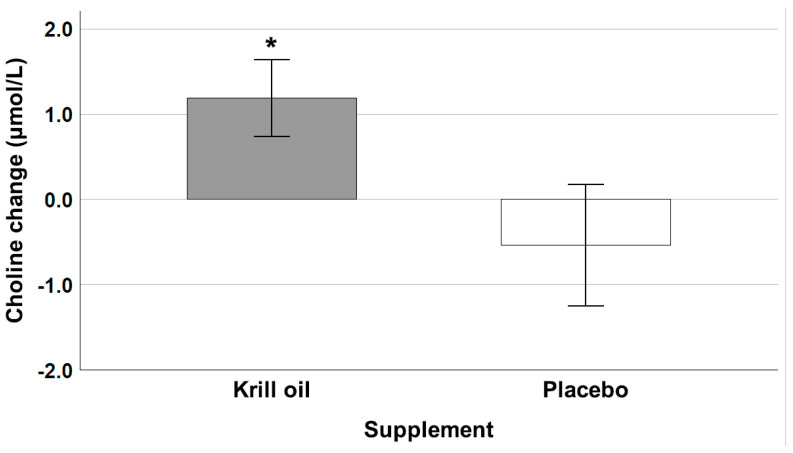
Mean (±SE) change in plasma choline concentration (µmol/L) from the end of the first exercise test (pre-supplementation) to the beginning of the second exercise test (post-supplementation) for the krill oil and placebo groups. * indicates a significant group difference (*p* < 0.05).

A follow-up *t*-test further revealed that the krill oil group filled up the choline pool significantly more over time after exercise relative to the placebo group from the end of the first pre-supplementation exercise test (marked in Figure 3 with #1 and §1 for krill oil and placebo, respectively) to the beginning of the post-supplementation exercise test (marked in Figure 3 with #2 and §2 for krill oil and placebo, respectively) (t19 = 2.15, *p* = 0.04), see Figure 4. 

### 3.6. Total Antioxidant Capacity

A mixed-design ANOVA revealed a significant main effect of exercise (F1,20 = 20.29, *p* < 0.001) due to the fact that TAC was reduced as a result of exercise in the krill oil and placebo groups prior to the supplementation period (Table 6). There were no other significant main or interaction effects. Interestingly, an inspection of Table 6 reveals that TAC post-supplementation following exercise was only reduced in the placebo group; however, the decrease was greater in the placebo group relative to the krill oil group (a mean reduction in the placebo group of 16% from 179.0 to 141.5 versus a mean reduction of 0.2% from 149.2 to 137.2 in the krill oil group). However, this group difference in mean TAC change from pre- to post-exercise was not significant (*p* = 0.35). 

## 4. Discussion

PT is a demanding fitness discipline that shows increasing popularity but because of high-intensity training sessions, it might benefit from nutritional optimization. The study showed three areas of interest that can be influenced by dietary krill oil supplementation, i.e., (a) enhanced choline availability, (b) optimized HS-Omega-3 Index and (c) improved TAC.

(a) Enhanced choline availability

A depletion of circulating choline has previously been described in endurance athletes with stronger observed decreases after strenuous and prolonged physical training or competition events [24]. It is therefore interesting to note, that one hour of intense PT can also lead to a 7.3% reduction in plasma choline concentrations when looked at all pre-supplementation participants pooled together. This is a significant decrease, albeit not in the range of marathon runners who showed a post-race drop of 40% [25] or Norseman triathletes with a 34% reduction [31]. It also should be pointed out that the post-exercise circulating choline concentrations, which in this study ranged between 6.7 and 13.6 µmol/L, were still within the range of healthy persons (7–12 µmol/L [61,62,63]).

Nevertheless, 12 weeks of krill oil supplementation increased plasma choline concentrations in the krill oil group (1.19) in comparison to a decrease in the placebo group (−0.53). This suggests that without optimal oral choline intake, the body cannot fully recover and will further deplete choline stores [22]. It was suggested that for activities that reduce circulating choline levels below normal, oral choline supplementation might increase endurance performance [24], since choline is needed to produce acetylcholine for optimal muscle performance [25,64]. Assessing performance was not part of this pilot study and it is unclear if a 7.3% drop in circulating choline concentrations can have an effect on power performance but could be of interest in a follow-up study. Another way that low choline levels can reduce sports performance, besides reduced work capacity, is impaired cognitive function. The assessment of cognitive function was part of the original protocol for this study, but due to the current COVID-19 pandemic, could not be included in the test parameters and is kept for a next study.

(b) Optimized HS-Omega-3 Index

In the recruitment for this study, we observed that the prevalence for an HS-Omega-3 Index above 8% was only 17.9%, while 37.9% of the subjects were below 6%. The study population is considered to be physically fit and healthy, yet more than 80% of the subjects were below the optimal HS-Omega-3 Index range of 8–11%, which may underlie great risk for potential cardiovascular incidents. On the other hand, krill oil supplementation with 2.5 g/d provides 550 mg of EPA and DHA, which is more than the 250 mg/d that are recommended as an adequate intake for the maintenance of general cardiovascular health by the European Food Safety Agency (EFSA) [65]. The krill oil dose given for twelve weeks increased the HS-Omega-3 Index in power athletes from 4.82 to 6.77%, whereas there was no increase in the placebo group, which confirmed the correct study product intake. 

Even though 6.77% is slightly below the optimal target of 8%, changes from 3.58 to 6.87% and from 3.3 to 5% were previously associated with a decreased risk for sudden cardiac death in a prospective cohort study by about 80% [66] and by a 70% reduction for risk of primary cardiac arrest in a case-control study [67]. Such changes might therefore be of importance, in particular with respect to athletes who may have an elevated risk for sudden cardiac death when compared to the general population [68,69].

Dietary n-3 intake, sex, age, body mass index, alcohol consumption, smoking and the chemical form of n-3 PUFA can all influence the HS-Omega-3 Index [70]. In addition, there is a link between physical activity and the HS-Omega-3 Index according to Davinelli et al., who identified a gradual decrease of the HS-Omega-3 Index with a higher weekly running distance [71]. Metabolic adaptations after intense training can change n-3 and n-6 levels in membranes [71,72] and athletes are known to have lower n-3 blood levels [44,71,73,74,75,76]. Athletes may therefore profit from nutritional interventions that supply extra EPA and DHA to improve health and performance, reduce inflammation, speed up recovery, repair muscle damage, as well as to improve blood flow and RBC deformability to increase oxygen and nutrient delivery to exercising muscles [77]. Moreover, n-3 PUFAs can change the immune response in athletes by suppressing the pro-inflammatory effects of ARA [78] and the right balance between ARA and EPA is crucial in the regulation of inflammatory mediators and resistance to infection [79,80,81]. In fact, both the n-6/n-3 ratio (from 5.84 to 3.77%; *p* < 0.001) and the ARA/EPA ratio (from 50.72 to 13.61%; *p* < 0.001) were significantly reduced by krill oil supplementation.

(c) Improved total antioxidant capacity

It was encouraging to note that there was a tendency to a lower reduction in the TAC post-exercise in the krill oil (−8%) in comparison to the placebo group (−21%). However, significance was not reached, which might be explained by the low number of tested subjects and should be verified in a larger study setup. A low TAC is indicative of oxidative stress or increased susceptibility to oxidative damage. The lower reduction of the antioxidant capacity after supplementation with krill oil may indicate that tissues avoid depleting the total antioxidant reserves to neutralize oxidative stress caused by exercise and muscle microlesions. In other words, the possible protection of the higher levels of n-3 PUFAs and astaxanthin in the cell membranes may minimize cell injury and/or the antioxidant action of these nutrients limit the use of the internal TAC reserves. Hence, the results suggest that krill oil increases the capacity to scavenge free radicals; however, direct measurements of biomarkers of oxidative stress were not part of this study and should be addressed in the future to exclude a potential mismatch between in vitro and in vivo findings.

## 5. Conclusions

While this article cannot give answers to whether the combined administration of n-3 PUFAs, choline and astaxanthin in the form of krill oil will improve PT performance, it can nevertheless highlight the potential areas where these nutrients can be of benefit, i.e., HS-Omega-3 Index optimization before training, as well as choline and oxidative stress recovery after training. However, it is important to point out that this is a pilot study that included a low number of participants because of COVID-19 restrictions, which makes it best suited to detect interesting trends. Future studies should address the involvement of krill nutrients in improving high-volume muscular endurance workout performance.

## Figures and Tables

**Table 1:** Characteristics and nutritional composition of study products.

Product	Krill Oil	Placebo
Product Name	NKO^TM^ krill oil	Virgin olive oil
Manufacturer	Aker BioMarine, Norway	Lonza Group, Switzerland
Amount per capsule (mg)	500	500
Capsules per day	5	5
Weeks of administration	12	12
EPA/DHA (g/100 g)	16/6	<1/<1
Total phospholipids (g/100 g)	46	<1
Choline (g/100 g)	6	<1
Esterified astaxanthin (mg/kg)	691	<1

**Table 2:** Flowchart of study design.

	Time	Phase	Activity
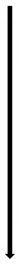	3 months	Subject selection and characterization	1. Subject selection according to eligibility criteria
			2. Subject characterization (medical examination, ECG, max exercise test, anthropometry, standard blood analysis)
	15 days	Group determination	
	1 day	First training day	3. Blood analysis:Omega-3 Index, TAC, inflammatory parameters
			4. Power training session (Heart rate, lactate, RPE)
			5. Blood analysis: TAC, inflammatory parameters
	12 weeks	Supplementation period: Placebo (*n* = 16) / Krill oil (*n* = 19)	
	1 day	Second training day	3. Blood analysis:Omega-3 Index, TAC, inflammatory parameters
			4. Power training session (Heart rate, lactate, RPE)
			5. Blood analysis: TAC, inflammatory parameters

**Table 3:** Characteristics of study participants (mean ± SD). Significant group difference *p* < 0.05.

Product	Krill Oil	Placebo
*n* (male/female)	19 (16/3)	16 (11/5)
Age (years)	32.5 ± 9.6	33.7 ± 8.0
BMI (m/kg^2^)	23.9 ± 2.4	24.3 ± 2.7
Body fat (%)	11.0 ± 2.6	11.2 ± 2.6
Resting heart rate (bpm)	65.9 ± 9.55	70.8 ± 13.7
Maximum heart rate (bpm)	183.6 ± 11.9	182.9 ± 8.9
Maximal oxygen consumption (VO_2max_ ml/min/kg)	47.9 ± 5.6	44.9 ± 4.9
Heart rate after 1 min of recovery	149 ± 15	149 ± 18

**Table 4:** Changes in fatty acid composition pre-supplementation to post-supplementation by supplement type, krill oil or placebo. Superscripts denote a significant difference in within-group (row-wise) values at * *p* < 0.01, ** *p* < 0.001. Letters denote between-group differences (krill oil vs. placebo) post-supplementation, where ^a^ (*p* < 0.05), ^b^ (*p* < 0.001) indicate significantly greater mean values.

Fatty Acid (%)		Suppl.	Pre	Post	Fatty Acid (%)	Suppl.	Pre	Post
Polyunsaturated	18:3 n-3 (ALA)	Krill oil	0.08	0.08	∑Trans	Krill oil	0.61	0.58
		Placebo	0.08	0.08		Placebo	0.61	0.59
	20:5 n-3 (EPA)	Krill oil	0.41	1.34 **^b^	∑Saturated	Krill oil	38.84	38.69
		Placebo	0.44	0.40		Placebo	39.24	38.96
	22:5 n-3 (DPA)	Krill oil	1.60	2.46 **^b^	∑Monounsaturated	Krill oil	18.29	18.13
		Placebo	1.65	1.65		Placebo	17.90	17.96
	22:6 n-3 (DHA)	Krill oil	4.41	5.31 **^a^				
		Placebo	4.72	4.71				
	18:2 n-6 (linoleic)	Krill oil	13.41	13.05				
		Placebo	13.68	13.30				
	20:4 n-6 (ARA)	Krill oil	15.76	15.10 *				
		Placebo	15.76	16.24 ^a^				
	∑n-3	Krill oil	6.51	9.18 **^b^				
		Placebo	6.90	6.83				
	∑n-6	Krill oil	35.76	33.42 **				
		Placebo	35.36	35.66 ^b^				
	n-6/n-3	Krill oil	5.84	3.77 **				
		Placebo	5.27	5.37 ^b^				
	ARA/EPA	Krill oil	50.72	13.61 **				
		Placebo	41.33	48.41 ^b^				

**Table 5:** Exercise training session load described by HR 20/40 (heart rate means during the last 60 s of the 20- or 40-min periods), lactate (at the 3rd minute of rest of every period) and rating perceived exertion (RPE, Borg scale with 10 items at the end of the training session).

	First Training Session		Second Training Session	
	Krill Oil	Placebo	Krill Oil	Placebo
Subject number	14	8	14	8
HR 20 (beats/min)	173 (9)	171 (12)	168 (11)	166 (13)
Lactate (mM/mL)	8.2 (2.2)	8.5 (3.1)	8.8 (2.1)	7.3 (2.3)
HR 40 (beats/min)	184 (7)	179 (9)	179 (8)	178 (10)
Lactate (mM/mL)	11.6 (1.8)	12.3 (3.1)	12.8 (2.0)	11.6 (3.3)
RPE	8.3 (0.7)	8.4 (0.6)	8.3 (0.9)	8.5 (0.7)

**Table 6:** Mean (±SD) total antioxidant capacity results (µM copper-reducing equivalent) pre- and post-exercise with and without supplementation with krill oil or placebo.

	Pre-Supplementation			Post-Supplementation		
	Before 1st Session	After 1st Session	Δ%1	Before 2nd Session	After 2nd Session	Δ%2
Placebo (*n* = 8)	186.0 (±24.9)	140.7 (±28.0)	−24.4 (±9.8)	179.0 (±58.5)	141.5 (±44.4)	−15.8 (±31.0)
Krill oil (*n* = 14)	158.8 (±34.6)	115.4 (±28.2)	−25.2 (±20.3)	149.2 (±45.8)	137.2 (±43.7)	−0.2 (±38.6)

## Data Availability

The raw data supporting the conclusions of this article will be made available by the authors, without undue reservation.

## References

[1] Butcher S.J., Judd T.B., Benko C.R., Horvey K.J., Pshyk A.D. (2015). Relative intensity of two types of CrossFit exercise: Acute circuit and high-intensity interval exercise. J. Fit. Res..

[2] Glassman G. (2010). The CrossFit training guide. Cross Fit J..

[3] Ben-Zeev T., Okun E. (2021). High-Intensity Functional Training: Molecular Mechanisms and Benefits. Neuromolecular Med..

[4] Falk Neto J.H., Kennedy M.D. (2019). The multimodal nature of high-intensity functional training: Potential applications to improve sport performance. Sports.

[5] Hayes L.D., Elliott B.T., Yasar Z., Bampouras T.M., Sculthorpe N.F., Sanal-Hayes N.E., Hurst C. (2021). High intensity interval training (HIIT) as a potential countermeasure for phenotypic characteristics of sarcopenia: A scoping review. Front. Physiol..

[6] Tibana R.A., De Sousa N.M.F. (2018). Are extreme conditioning programmes effective and safe? A narrative review of high-intensity functional training methods research paradigms and findings. BMJ Open Sport Exerc. Med..

[7] Banaszek A., Townsend J.R., Bender D., Vantrease W.C., Marshall A.C., Johnson K.D. (2019). The effects of whey vs. pea protein on physical adaptations following 8-weeks of high-intensity functional training (HIFT): A pilot study. Sports.

[8] Durkalec-Michalski K., Zawieja E.E., Podgórski T., Łoniewski I., Zawieja B.E., Warzybok M., Jeszka J. (2018). The effect of chronic progressive-dose sodium bicarbonate ingestion on CrossFit-like performance: A double-blind, randomized cross-over trial. PLoS ONE.

[9] Escobar K.A., Morales J., Vandusseldorp T.A. (2016). The effect of a moderately low and high carbohydrate intake on crossfit performance. Int. J. Exerc. Sci..

[10] Kramer S.J., Baur D.A., Spicer M.T., Vukovich M.D., Ormsbee M.J. (2016). The effect of six days of dietary nitrate supplementation on performance in trained CrossFit athletes. J. Int. Soc. Sports Nutr..

[11] Sadowska-Krępa E., Domaszewski P., Pokora I., Żebrowska A., Gdańska A., Podgórski T. (2019). Effects of medium-term green tea extract supplementation combined with CrossFit workout on blood antioxidant status and serum brain-derived neurotrophic factor in young men: A pilot study. J. Int. Soc. Sports Nutr..

[12] Stein J.A., Ramirez M., Heinrich K.M. (2020). Acute caffeine supplementation does not improve performance in trained CrossFit^®^ athletes. Sports.

[13] Urbina S., Hayward S., Outlaw J., Holt J., Burks B., Cox B., Faillace E., Stai B., Stone M., Wildman R. (2013). Performance and body composition effects of a pre-workout supplement and post-workout protein intake in trained crossfit individuals. J. Int. Soc. Sports Nutr..

[14] Gogojewicz A., Śliwicka E., Durkalec-Michalski K. (2020). Assessment of dietary intake and nutritional status in CrossFit-trained individuals: A descriptive study. Int. J. Environ. Res. Public Health.

[15] Jensen H.H., Batres-Marquez S.P., Carriquiry A., Schalinske K.L. (2007). Choline in the diets of the US population: NHANES, 2003–2004. FASEB J..

[16] Panel E.N. (2016). Scientific opinion on Dietary Reference Values for choline. EFSA J..

[17] Jäger R., Purpura M., Kingsley M. (2007). Phospholipids and sports performance. J. Int. Soc. Sports Nutr..

[18] Kohlmeier M., da Costa K.-A., Fischer L.M., Zeisel S.H. (2005). Genetic variation of folate-mediated one-carbon transfer pathway predicts susceptibility to choline deficiency in humans. Proc. Natl. Acad. Sci. USA.

[19] Buchman A.L., Jenden D., Roch M. (1999). Plasma free, phospholipid-bound and urinary free choline all decrease during a marathon run and may be associated with impaired performance. J. Am. Coll. Nutr..

[20] Conlay L., Sabounjian L., Wurtman R. (1992). Exercise and neuromodulators. Int. J. Sports Med..

[21] Piérard C., Béracochéa D., Pérès M., Jouanin J.-C., Liscia P., Satabin P., Martin S., Testylier G., Guézennec C.Y., Beaumont M. (2004). Declarative memory impairments following a military combat course: Parallel neuropsychological and biochemical investigations. Neuropsychobiology.

[22] da Costa K.-A., Badea M., Fischer L.M., Zeisel S.H. (2004). Elevated serum creatine phosphokinase in choline-deficient humans: Mechanistic studies in C2C12 mouse myoblasts. Am. J. Clin. Nutr..

[23] Klein J. (2000). Membrane breakdown in acute and chronic neurodegeneration: Focus on choline-containing phospholipids. J. Neural Transm..

[24] Penry J.T., Manore M.M. (2008). Choline: An important micronutrient for maximal endurance-exercise performance?. Int. J. Sport Nutr. Exerc. Metab..

[25] Conlay L., Wurtman R., Blusztajn K., Coviella I., Maher T., Evoniuk G. (1986). Decreased plasma choline concentrations in marathon runners. N. Engl. J. Med..

[26] von Allwörden H.N., Horn S., Kahl J., Feldheim W. (1993). The influence of lecithin on plasma choline concentrations in triathletes and adolescent runners during exercise. Eur. J. Appl. Physiol. Occup. Physiol..

[27] Beckham G., Mizuguchi S., Carter C., Sato K., Ramsey M., Lamont H., Hornsby G., Haff G., Stone M. (2013). Relationships of isometric mid-thigh pull variables to weightlifting performance. J. Sports Med. Phys. Fit..

[28] Burri L., Hoem N., Banni S., Berge K. (2012). Marine omega-3 phospholipids: Metabolism and biological activities. Int. J. Mol. Sci..

[29] Mödinger Y., Schön C., Wilhelm M., Hals P.-A. (2019). Plasma kinetics of choline and choline metabolites after a single dose of SuperbaBoostTM krill oil or choline bitartrate in healthy volunteers. Nutrients.

[30] Berge R.K., Ramsvik M.S., Bohov P., Svardal A., Nordrehaug J.E., Rostrup E., Bruheim I., Bjørndal B. (2015). Krill oil reduces plasma triacylglycerol level and improves related lipoprotein particle concentration, fatty acid composition and redox status in healthy young adults-a pilot study. Lipids Health Dis..

[31] Storsve A.B., Johnsen L., Nyborg C., Melau J., Hisdal J., Burri L. (2020). Effects of krill oil and race distance on serum choline and choline metabolites in triathletes: A field study. Front. Nutr..

[32] Calder P.C. (2015). Marine omega-3 fatty acids and inflammatory processes: Effects, mechanisms and clinical relevance. Biochim. Biophys. Acta (BBA) Mol. Cell Biol. Lipids.

[33] Harris W.S. (2008). The omega-3 index as a risk factor for coronary heart disease. Am. J. Clin. Nutr..

[34] Harris W.S., Von Schacky C. (2004). The Omega-3 Index: A new risk factor for death from coronary heart disease?. Prev. Med..

[35] Serhan C.N., Chiang N., Van Dyke T.E. (2008). Resolving inflammation: Dual anti-inflammatory and pro-resolution lipid mediators. Nat. Rev. Immunol..

[36] Simpson R.J., Campbell J.P., Gleeson M., Krüger K., Nieman D.C., Pyne D.B., Turner J.E., Walsh N.P. (2020). Can exercise affect immune function to increase susceptibility to infection?. Exerc. Immunol. Rev..

[37] Da Boit M., Hunter A.M., Gray S.R. (2017). Fit with good fat? The role of n-3 polyunsaturated fatty acids on exercise performance. Metabolism.

[38] Da Boit M., Mastalurova I., Brazaite G., McGovern N., Thompson K., Gray S.R. (2015). The effect of krill oil supplementation on exercise performance and markers of immune function. PLoS ONE.

[39] Smith G.I., Atherton P., Reeds D.N., Mohammed B.S., Rankin D., Rennie M.J., Mittendorfer B. (2011). Omega-3 polyunsaturated fatty acids augment the muscle protein anabolic response to hyperinsulinaemia–hyperaminoacidaemia in healthy young and middle-aged men and women. Clin. Sci..

[40] Georges J., Sharp M.H., Lowery R.P., Wilson J.M., Purpura M., Hornberger T.A., Harding F., Johnson J.H., Peele D.M., Jäger R. (2018). The effects of krill oil on mTOR signaling and resistance exercise: A pilot study. J. Nutr. Metab..

[41] Von Schacky C. (2014). Omega-3 index and cardiovascular health. Nutrients.

[42] Metcalf R.G., Cleland L.G., Gibson R.A., Roberts-Thomson K.C., Edwards J.R., Sanders P., Stuklis R., James M.J., Young G.D. (2010). Relation between blood and atrial fatty acids in patients undergoing cardiac bypass surgery. Am. J. Clin. Nutr..

[43] von Schacky C. (2011). The Omega-3 Index as a risk factor for cardiovascular diseases. Prostaglandins Other Lipid Mediat..

[44] Drobnic F., Rueda F., Pons V., Banquells M., Cordobilla B., Domingo J.C. (2017). Erythrocyte omega-3 fatty acid content in elite athletes in response to omega-3 supplementation: A dose-response pilot study. J. Lipids.

[45] Kris-Etherton P.M., Harris W.S., Appel L.J. (2002). Fish consumption, fish oil, omega-3 fatty acids, and cardiovascular disease. Circulation.

[46] Burdge G. (2004). α-Linolenic acid metabolism in men and women: Nutritional and biological implications. Curr. Opin. Clin. Nutr. Metab. Care.

[47] Alessio H.M., Hagerman A.E., Fulkerson B.K., Ambrose J., Rice R.E., Wiley R.L. (2000). Generation of reactive oxygen species after exhaustive aerobic and isometric exercise. Med. Sci. Sports Exerc..

[48] Finaud J., Lac G., Filaire E. (2006). Oxidative stress. Sports Med..

[49] Reid M.B. (2016). Redox interventions to increase exercise performance. J. Physiol..

[50] Bouzid M.A., Filaire E., Matran R., Robin S., Fabre C. (2018). Lifelong voluntary exercise modulates age-related changes in oxidative stress. Int. J. Sports Med..

[51] Margonis K., Fatouros I.G., Jamurtas A.Z., Nikolaidis M.G., Douroudos I., Chatzinikolaou A., Mitrakou A., Mastorakos G., Papassotiriou I., Taxildaris K. (2007). Oxidative stress biomarkers responses to physical overtraining: Implications for diagnosis. Free Radic. Biol. Med..

[52] Skarpańska-Stejnborn A., Pilaczyńska-Szcześniak Ł., Basta P., Foriasz J., Arlet J. (2015). Effects of supplementation with Neptune krill oil (euphasia superba) on selected redox parameters and pro-inflammatory markers in athletes during exhaustive exercise. J. Hum. Kinet..

[53] Wen C., Jiang M., Huang W., Liu S. (2020). Antarctic Krill Oil Attenuates Oxidative Stress via the KEAP1-NRF2 Signaling in Patients with Coronary Heart Disease. Evid. Based Complement. Altern. Med..

[54] Miki W. (1991). Biological functions and activities of animal carotenoids. Pure Appl. Chem..

[55] Djordjevic B., Baralic I., Kotur-Stevuljevic J., Stefanovic A., Ivanisevic J., Radivojevic N., Andjelkovic M., Dikic N. (2012). Effect of astaxanthin supplementation on muscle damage and oxidative stress markers in elite young soccer players. J. Sports Med. Phys. Fit..

[56] Earnest C.P., Lupo M., White K., Church T. (2011). Effect of astaxanthin on cycling time trial performance. Int. J. Sports Med..

[57] Malmsten C., Lignell A. (2008). Dietary Supplementation with Astaxanthin-Rich Algal Meal Improves Strength Endurance–A Double Blind Placebo Controlled Study on Male Students. Carotenoid Sci..

[58] Foster C., Florhaug J.A., Franklin J., Gottschall L., Hrovatin L.A., Parker S., Doleshal P., Dodge C. (2001). A new approach to monitoring exercise training. J. Strength Cond. Res..

[59] Faulkner J.A. (1968). Physiology of Swimming and Diving.

[60] Yuhasz M.S. (1962). The Effects of Sports Training on Body Fat in Man with Predictions of Optimal Body Weight.

[61] Midttun Ø., Kvalheim G., Ueland P.M. (2013). High-throughput, low-volume, multianalyte quantification of plasma metabolites related to one-carbon metabolism using HPLC-MS/MS. Anal. Bioanal. Chem..

[62] Cho C.E., Taesuwan S., Malysheva O.V., Bender E., Yan J., Caudill M.A. (2016). Choline and one-carbon metabolite response to egg, beef and fish among healthy young men: A short-term randomized clinical study. Clin. Nutr. Exp..

[63] Zeisel S.H., Da Costa K.A., Franklin P.D., Alexander E.A., Lamont J.T., Sheard N.F., Beiser A. (1991). Choline, an essential nutrient for humans. FASEB J..

[64] Kanter M.M., Williams M.H. (1995). Antioxidants, carnitine, and choline as putative ergogenic aids. Int. J. Sport Nutr. Exerc. Metab..

[65] EFSA Panel on Dietetic Products, Nutrition and Allergies (NDA) (2011). Scientific Opinion on the substantiation of health claims related to docosahexaenoic acid (DHA), eicosapentaenoic acid (EPA) and brain, eye and nerve development (ID 501, 513, 540), maintenance of normal brain function (ID 497, 501, 510, 513, 519, 521, 534, 540, 688, 1323, 1360, 4294), maintenance of normal vision (ID 508, 510, 513, 519, 529, 540, 688, 2905, 4294), maintenance of normal cardiac function (ID 510, 688, 1360),“maternal health; pregnancy and nursing”(ID 514),“to fulfil increased omega-3 fatty acids need during pregnancy”(ID 539),“skin and digestive tract epithelial cells maintenance”(ID 525), enhancement of mood (ID 536),“membranes cell structure”(ID 4295),“anti-inflammatory action”(ID 4688) and maintenance of normal blood LDL-cholesterol concentrations (ID 4719) pursuant to Article 13 (1) of Regulation (EC) No 1924/2006. EFSA J..

[66] Albert C.M., Campos H., Stampfer M.J., Ridker P.M., Manson J.E., Willett W.C., Ma J. (2002). Blood levels of long-chain n–3 fatty acids and the risk of sudden death. N. Engl. J. Med..

[67] Siscovick D.S., Raghunathan T., King I., Weinmann S., Wicklund K.G., Albright J., Bovbjerg V., Arbogast P., Smith H., Kushi L.H. (1995). Dietary intake and cell membrane levels of long-chain n-3 polyunsaturated fatty acids and the risk of primary cardiac arrest. JAMA.

[68] Harmon K.G., Asif I.M., Maleszewski J.J., Owens D.S., Prutkin J.M., Salerno J.C., Zigman M.L., Ellenbogen R., Rao A.L., Ackerman M.J. (2015). Incidence, cause, and comparative frequency of sudden cardiac death in national collegiate athletic association athletes: A decade in review. Circulation.

[69] Schmied C., Borjesson M. (2014). Sudden cardiac death in athletes. J. Intern. Med..

[70] de Groot R.H., Emmett R., Meyer B.J. (2019). Non-dietary factors associated with n-3 long-chain PUFA levels in humans–A systematic literature review. Br. J. Nutr..

[71] Davinelli S., Corbi G., Righetti S., Casiraghi E., Chiappero F., Martegani S., Pina R., De Vivo I., Simopoulos A.P., Scapagnini G. (2019). Relationship between distance run per week, omega-3 index, and arachidonic acid (AA)/Eicosapentaenoic acid (EPA) ratio: An observational retrospective study in non-elite runners. Front. Physiol..

[72] Tepsic J., Vucic V., Arsic A., Blazencic-Mladenovic V., Mazic S., Glibetic M. (2009). Plasma and erythrocyte phospholipid fatty acid profile in professional basketball and football players. Eur. J. Appl. Physiol..

[73] Anzalone A., Carbuhn A., Jones L., Gallop A., Smith A., Johnson P., Swearingen L., Moore C., Rimer E., McBeth J. (2019). The omega-3 index in National Collegiate Athletic Association division I collegiate football athletes. J. Athl. Train..

[74] Ritz P.P., Rogers M.B., Zabinsky J.S., Hedrick V.E., Rockwell J.A., Rimer E.G., Kostelnik S.B., Hulver M.W., Rockwell M.S. (2020). Dietary and biological assessment of the Omega-3 status of collegiate athletes: A cross-sectional analysis. PLoS ONE.

[75] von Schacky C., Kemper M., Haslbauer R., Halle M. (2014). Low omega-3 index in 106 German elite winter endurance athletes: A pilot study. Int. J. Sport Nutr. Exerc. Metab..

[76] Wilson P.B., Madrigal L.A. (2016). Associations between whole blood and dietary omega-3 polyunsaturated fatty acid levels in collegiate athletes. Int. J. Sport Nutr. Exerc. Metab..

[77] Tiryaki-Sönmez G., Schoenfeld B., Vatansever-Ozen S. (2011). Omega-3 fatty acids and exercise: A review of their combined effects on body composition and physical performance. Biomed. Hum. Kinet..

[78] Simopoulos A.P. (2008). The importance of the omega-6/omega-3 fatty acid ratio in cardiovascular disease and other chronic diseases. Exp. Biol. Med..

[79] Gleeson M. (2007). Immune function in sport and exercise. J. Appl. Physiol..

[80] Nieman D.C. (2000). Exercise effects on systemic immunity. Immunol. Cell Biol..

[81] Ninomiya T., Nagata M., Hata J., Hirakawa Y., Ozawa M., Yoshida D., Ohara T., Kishimoto H., Mukai N., Fukuhara M. (2013). Association between ratio of serum eicosapentaenoic acid to arachidonic acid and risk of cardiovascular disease: The Hisayama Study. Atherosclerosis.

